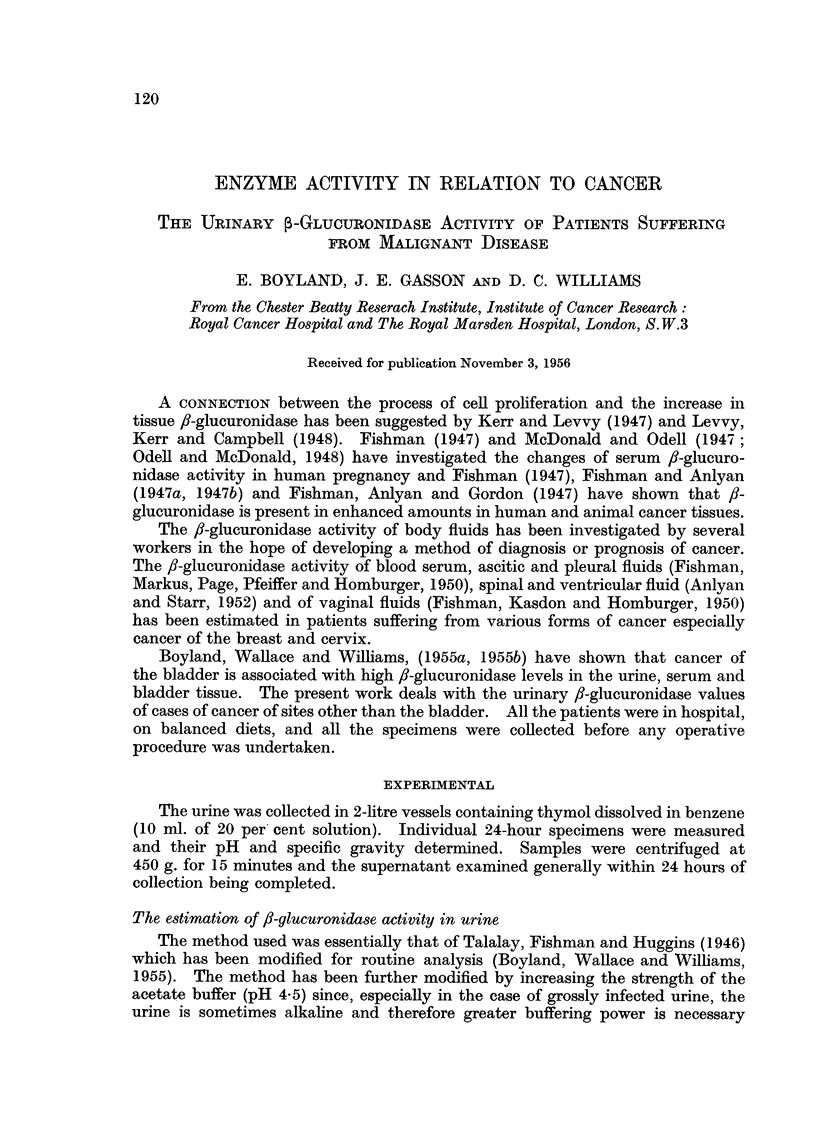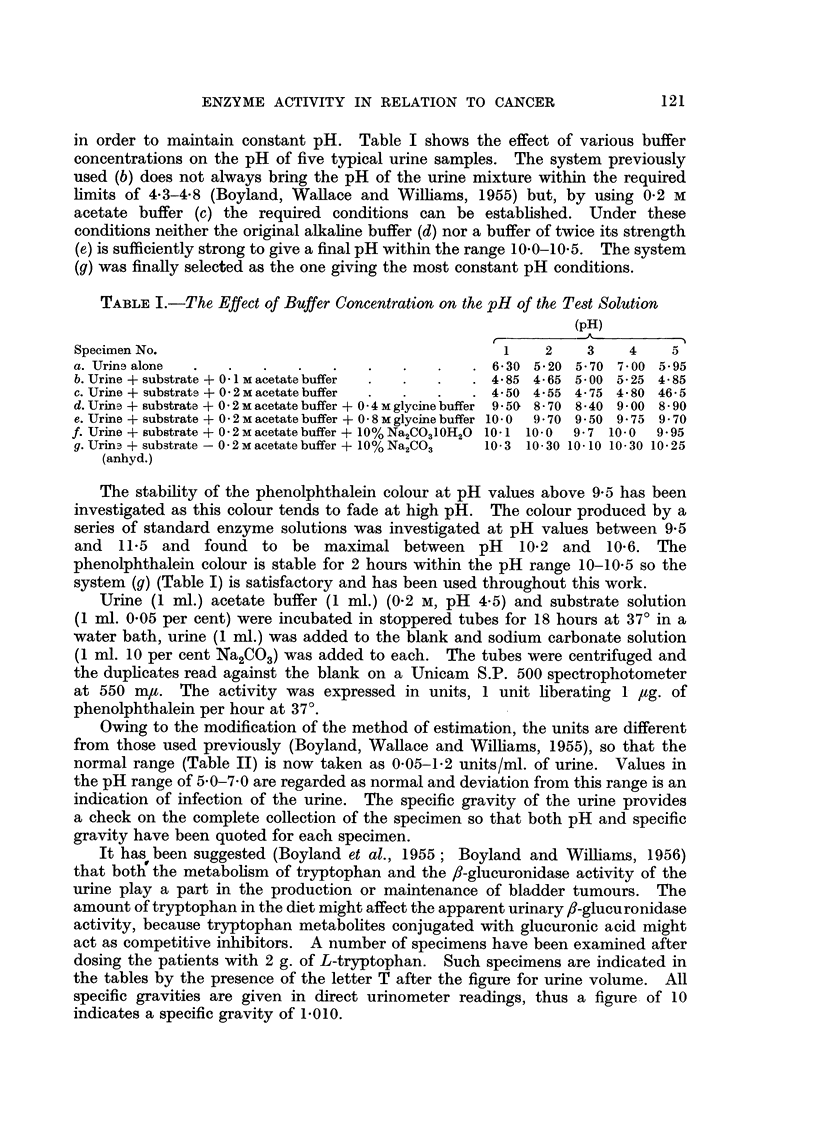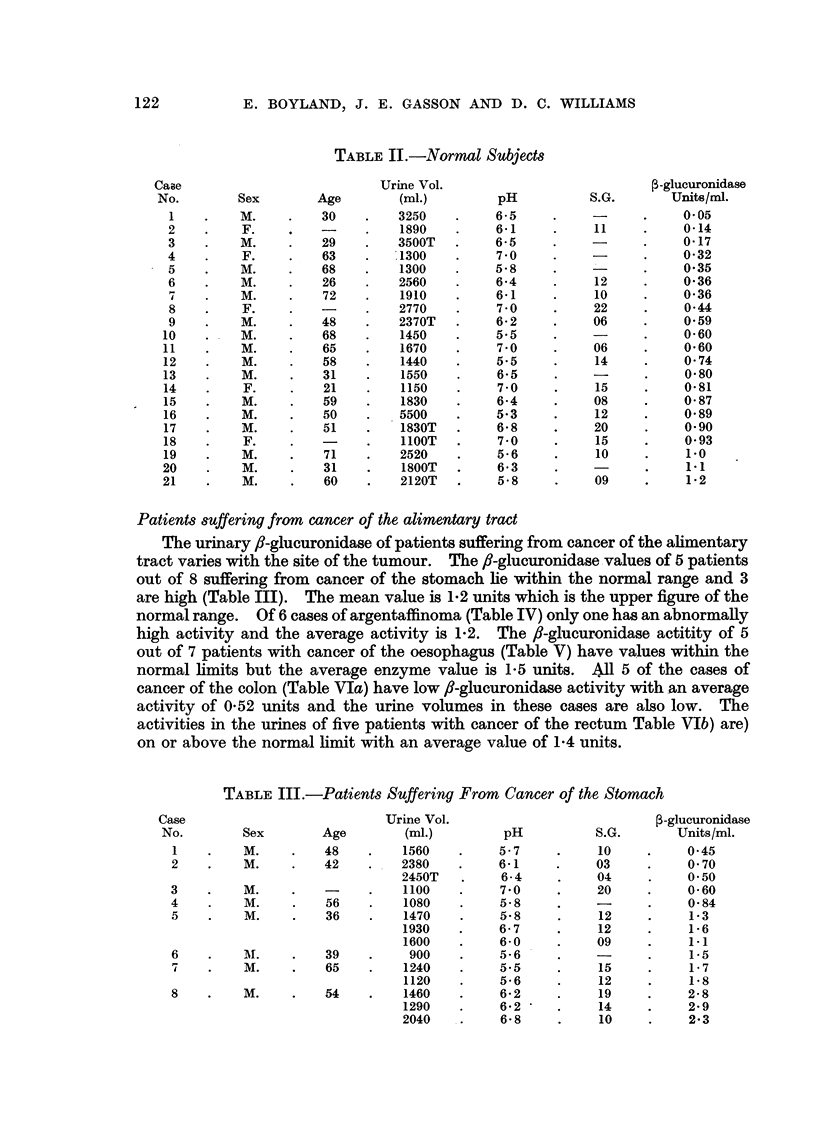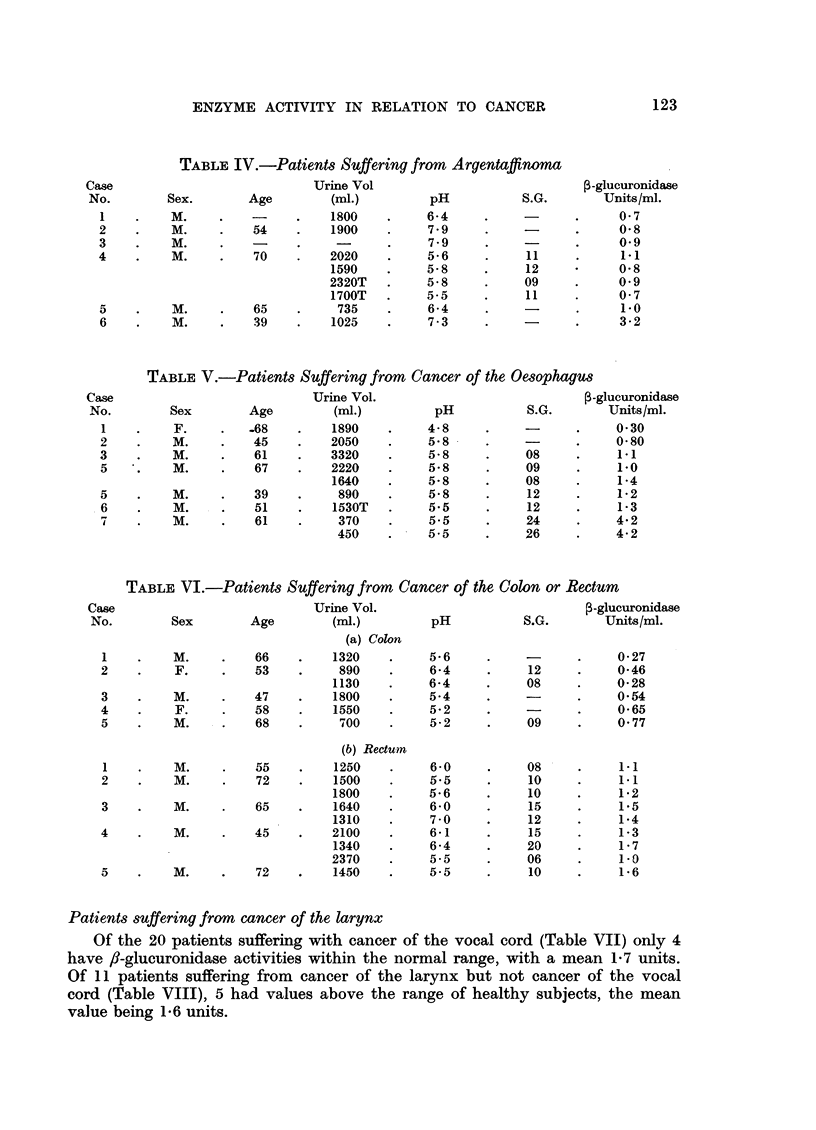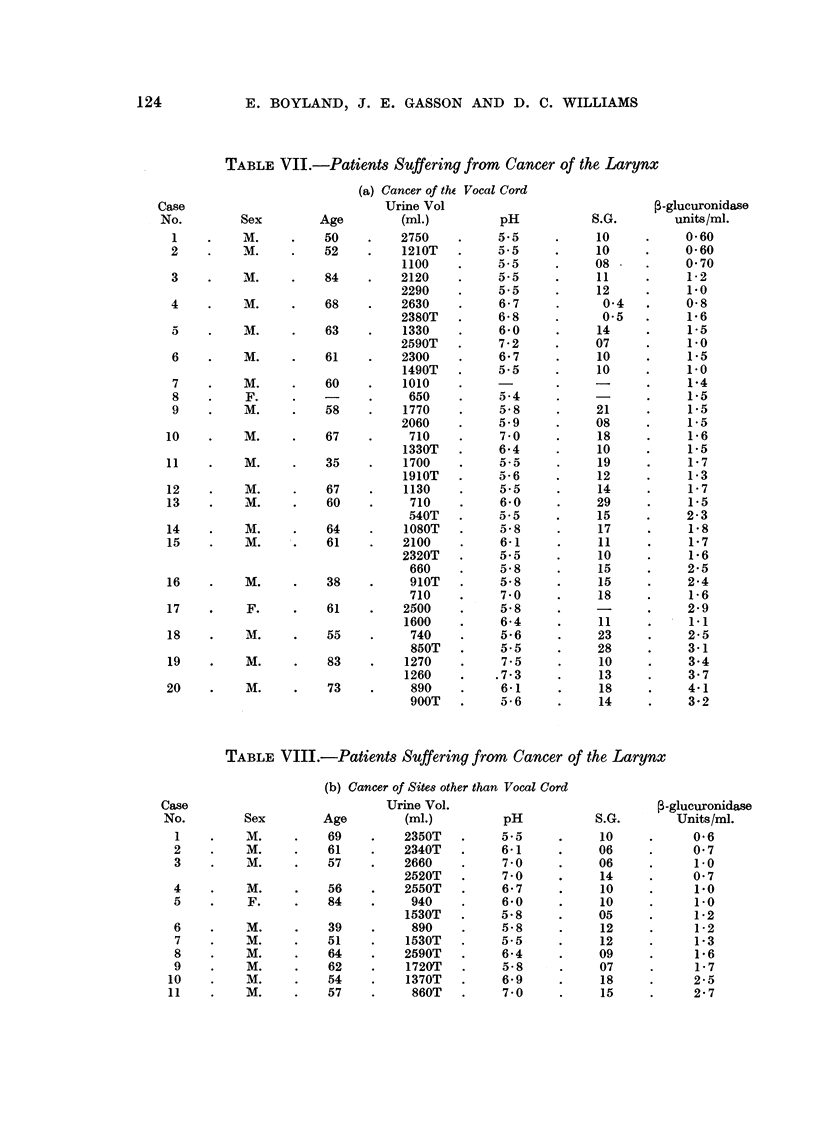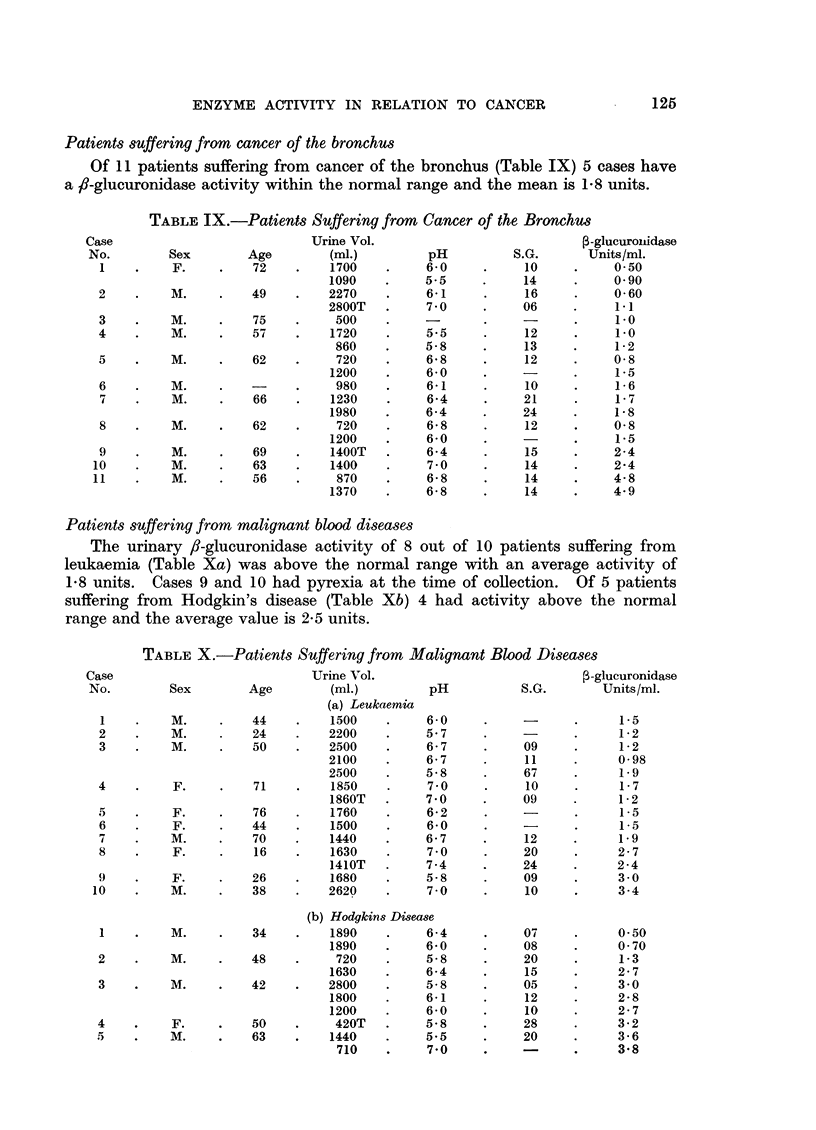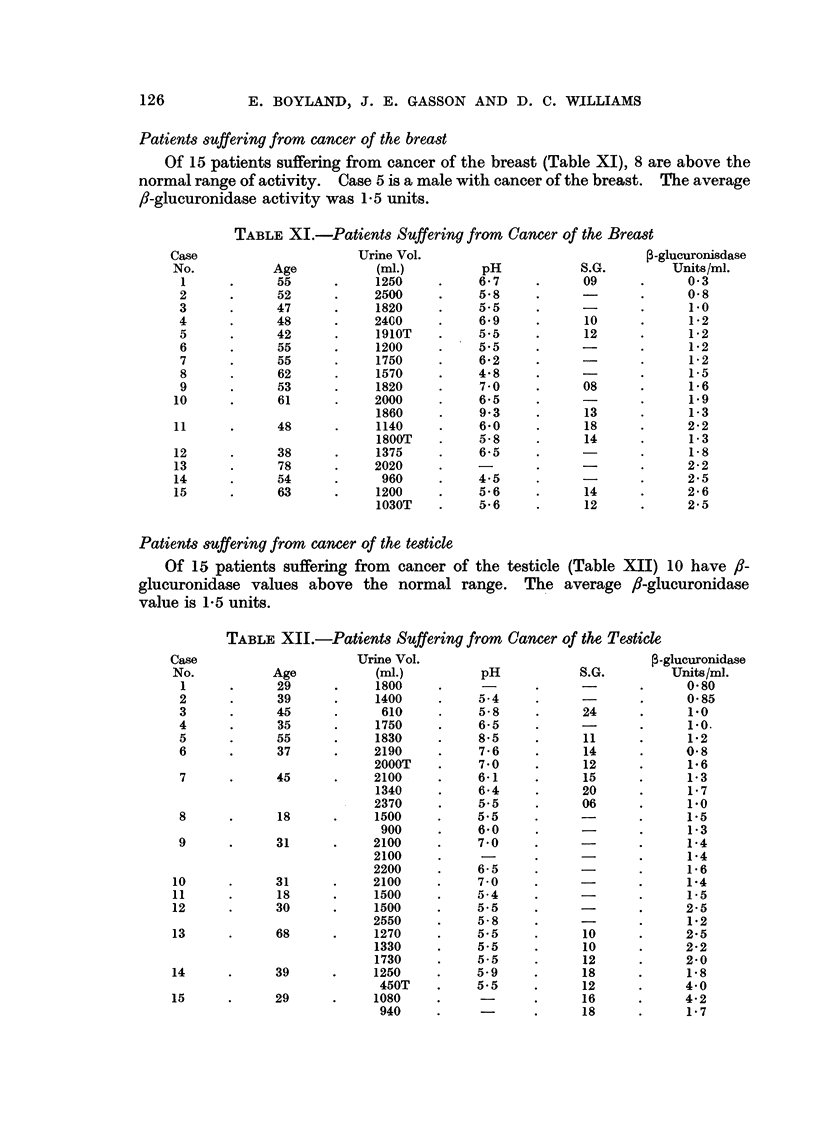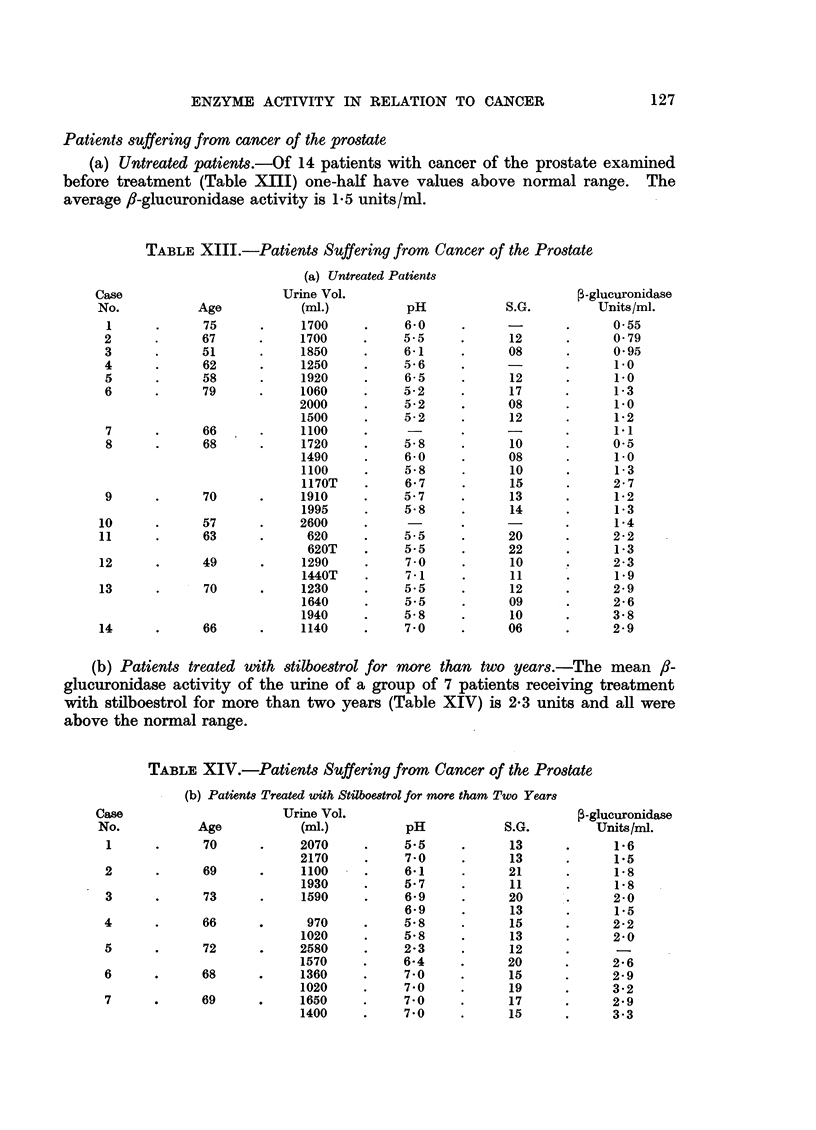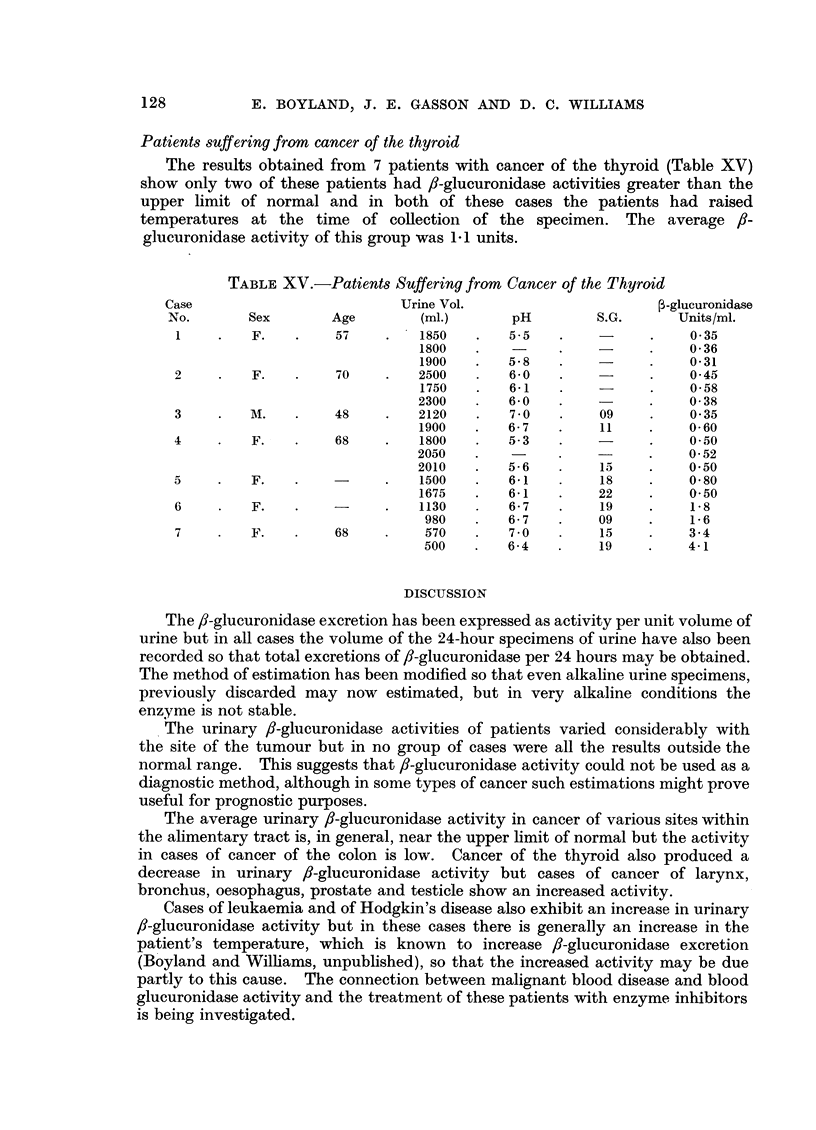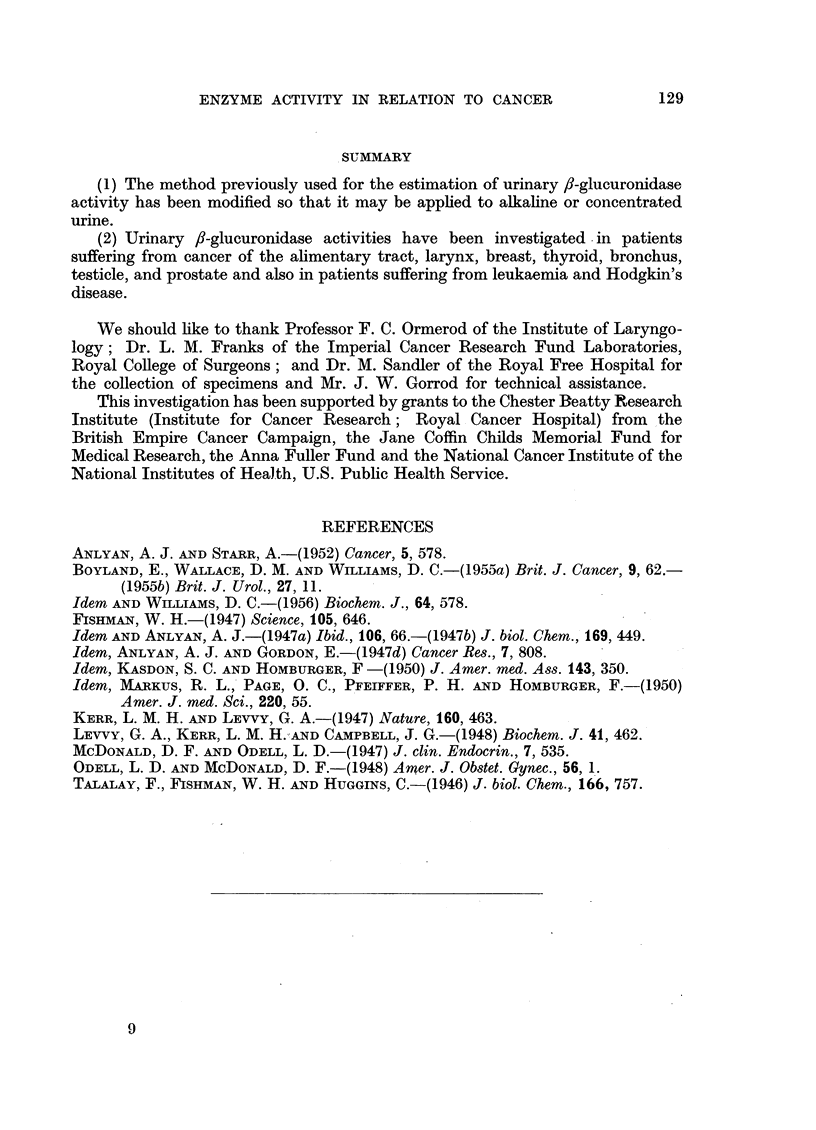# Enzyme Activity in Relation to Cancer

**DOI:** 10.1038/bjc.1957.18

**Published:** 1957-03

**Authors:** E. Boyland, J. E. Gasson, D. C. Williams


### ENZYME ACTIVITY IN RELATION TO CANCER

### THE URINARY β-GLUCURONIDASE ACTIVITY OF PATIENTS SUFFERING FROM MALIGNANT DISEASE

E. BOYLAND, J. E. GASSON AND D. C. WILLIAMS

*From the Chester Beatty Reserach Institute, Institute of Cancer Research : Royal Cancer Hospital and The Royal Marsden Hospital, London, S.W.3*

Received for publication November 3, 1956

A CONNECTION between the process of cell proliferation and the increase in tissue β-glucuronidase has been suggested by Kerr and Levvy (1947) and Levvy, Kerr and Campbell (1948). Fishman (1947) and McDonald and Odell (1947 ; Odell and McDonald, 1948) have investigated the changes of serum β-glucuronidase activity in human pregnancy and Fishman (1947), Fishman and Anlyan (1947*a*, 1947*b*) and Fishman, Anlyan and Gordon (1947) have shown that β-glucuronidase is present in enhanced amounts in human and animal cancer tissues.

The β-glucuronidase activity of body fluids has been investigated by several workers in the hope of developing a method of diagnosis or prognosis of cancer. The β-glucuronidase activity of blood serum, ascitic and pleural fluids (Fishman, Markus, Page, Pfeiffer and Homburger, 1950), spinal and ventricular fluid (Anlyan and Starr, 1952) and of vaginal fluids (Fishman, Kasdon and Homburger, 1950) has been estimated in patients suffering from various forms of cancer especially cancer of the breast and cervix.

Boyland, Wallace and Williams, (1955*a*, 1955*b*) have shown that cancer of the bladder is associated with high β-glucuronidase levels in the urine, serum and bladder tissue. The present work deals with the urinary β-glucuronidase values of cases of cancer of sites other than the bladder. All the patients were in hospital, on balanced diets, and all the specimens were collected before any operative procedure was undertaken.

### EXPERIMENTAL

The urine was collected in 2-litre vessels containing thymol dissolved in benzene (10 ml. of 20 per cent solution). Individual 24-hour specimens were measured and their pH and specific gravity determined. Samples were centrifuged at 450 g. for 15 minutes and the supernatant examined generally within 24 hours of collection being completed.

#### *The estimation of β-glucuronidase activity in urine*

The method used was essentially that of Talalay, Fishman and Huggins (1946) which has been modified for routine analysis (Boyland, Wallace and Williams, 1955). The method has been further modified by increasing the strength of the acetate buffer (pH 4·5) since, especially in the case of grossly infected urine, the urine is sometimes alkaline and therefore greater buffering power is necessary in order to maintain constant pH. Table I shows the effect of various buffer concentrations on the pH of five typical urine samples. The system previously used (*b*) does not always bring the pH of the urine mixture within the required limits of 4·3–4·8 (Boyland, Wallace and Williams, 1955) but, by using 0·2 M acetate buffer (*c*) the required conditions can be established. Under these conditions neither the original alkaline buffer (*d*) nor a buffer of twice its strength (*e*) is sufficiently strong to give a final pH within the range 10·0–10·5. The system (*g*) was finally selected as the one giving the most constant pH conditions.

TABLE I.—*The Effect of Buffer Concentration on the pH of the Test Solution*

| Specimen No. | (pH) 1 | 2 | 3 | 4 | 5 |
|---|---|---|---|---|---|
| *a*. Urine alone | 6·30 | 5·20 | 5·70 | 7·00 | 5·95 |
| *b*. Urine + substrate + 0·1 M acetate buffer | 4·85 | 4·65 | 5·00 | 5·25 | 4·85 |
| *c*. Urine + substrate + 0·2 M acetate buffer | 4·50 | 4·55 | 4·75 | 4·80 | 46·5 |
| *d*. Urine + substrate + 0·2 M acetate buffer + 0·4 M glycine buffer | 9·50 | 8·70 | 8·40 | 9·00 | 8·90 |
| *e*. Urine + substrate + 0·2 M acetate buffer + 0·8 M glycine buffer | 10·0 | 9·70 | 9·50 | 9·75 | 9·70 |
| *f*. Urine + substrate + 0·2 M acetate buffer + 10% $Na_2CO_3 10H_2O$ | 10·1 | 10·0 | 9·7 | 10·0 | 9·95 |
| *g*. Urine + substrate – 0·2 M acetate buffer + 10% $Na_2CO_3$ (anhyd.) | 10·3 | 10·30 | 10·10 | 10·30 | 10·25 |

The stability of the phenolphthalein colour at pH values above 9·5 has been investigated as this colour tends to fade at high pH. The colour produced by a series of standard enzyme solutions was investigated at pH values between 9·5 and 11·5 and found to be maximal between pH 10·2 and 10·6. The phenolphthalein colour is stable for 2 hours within the pH range 10–10·5 so the system (*g*) (Table I) is satisfactory and has been used throughout this work.

Urine (1 ml.) acetate buffer (1 ml.) (0·2 M, pH 4·5) and substrate solution (1 ml. 0·05 per cent) were incubated in stoppered tubes for 18 hours at 37° in a water bath, urine (1 ml.) was added to the blank and sodium carbonate solution (1 ml. 10 per cent $Na_2CO_3$) was added to each. The tubes were centrifuged and the duplicates read against the blank on a Unicam S.P. 500 spectrophotometer at 550 m$\mu$. The activity was expressed in units, 1 unit liberating 1 $\mu$g. of phenolphthalein per hour at 37°.

Owing to the modification of the method of estimation, the units are different from those used previously (Boyland, Wallace and Williams, 1955), so that the normal range (Table II) is now taken as 0·05–1·2 units/ml. of urine. Values in the pH range of 5·0–7·0 are regarded as normal and deviation from this range is an indication of infection of the urine. The specific gravity of the urine provides a check on the complete collection of the specimen so that both pH and specific gravity have been quoted for each specimen.

It has been suggested (Boyland *et al.*, 1955; Boyland and Williams, 1956) that both the metabolism of tryptophan and the $\beta$-glucuronidase activity of the urine play a part in the production or maintenance of bladder tumours. The amount of tryptophan in the diet might affect the apparent urinary $\beta$-glucuronidase activity, because tryptophan metabolites conjugated with glucuronic acid might act as competitive inhibitors. A number of specimens have been examined after dosing the patients with 2 g. of *L*-tryptophan. Such specimens are indicated in the tables by the presence of the letter T after the figure for urine volume. All specific gravities are given in direct urinometer readings, thus a figure of 10 indicates a specific gravity of 1·010.

TABLE II.—*Normal Subjects*

| Case No. | Sex | Age | Urine Vol. (ml.) | pH | S.G. | β-glucuronidase Units/ml. |
|---|---|---|---|---|---|---|
| 1 | M. | 30 | 3250 | 6·5 | — | 0·05 |
| 2 | F. | — | 1890 | 6·1 | 11 | 0·14 |
| 3 | M. | 29 | 3500T | 6·5 | — | 0·17 |
| 4 | F. | 63 | 1300 | 7·0 | — | 0·32 |
| 5 | M. | 68 | 1300 | 5·8 | — | 0·35 |
| 6 | M. | 26 | 2560 | 6·4 | 12 | 0·36 |
| 7 | M. | 72 | 1910 | 6·1 | 10 | 0·36 |
| 8 | F. | — | 2770 | 7·0 | 22 | 0·44 |
| 9 | M. | 48 | 2370T | 6·2 | 06 | 0·59 |
| 10 | M. | 68 | 1450 | 5·5 | — | 0·60 |
| 11 | M. | 65 | 1670 | 7·0 | 06 | 0·60 |
| 12 | M. | 58 | 1440 | 5·5 | 14 | 0·74 |
| 13 | M. | 31 | 1550 | 6·5 | — | 0·80 |
| 14 | F. | 21 | 1150 | 7·0 | 15 | 0·81 |
| 15 | M. | 59 | 1830 | 6·4 | 08 | 0·87 |
| 16 | M. | 50 | 5500 | 5·3 | 12 | 0·89 |
| 17 | M. | 51 | 1830T | 6·8 | 20 | 0·90 |
| 18 | F. | — | 1100T | 7·0 | 15 | 0·93 |
| 19 | M. | 71 | 2520 | 5·6 | 10 | 1·0 |
| 20 | M. | 31 | 1800T | 6·3 | — | 1·1 |
| 21 | M. | 60 | 2120T | 5·8 | 09 | 1·2 |

*Patients suffering from cancer of the alimentary tract*

The urinary β-glucuronidase of patients suffering from cancer of the alimentary tract varies with the site of the tumour. The β-glucuronidase values of 5 patients out of 8 suffering from cancer of the stomach lie within the normal range and 3 are high (Table III). The mean value is 1·2 units which is the upper figure of the normal range. Of 6 cases of argentaffinoma (Table IV) only one has an abnormally high activity and the average activity is 1·2. The β-glucuronidase actitity of 5 out of 7 patients with cancer of the oesophagus (Table V) have values within the normal limits but the average enzyme value is 1·5 units. All 5 of the cases of cancer of the colon (Table VI*a*) have low β-glucuronidase activity with an average activity of 0·52 units and the urine volumes in these cases are also low. The activities in the urines of five patients with cancer of the rectum Table VI*b*) are) on or above the normal limit with an average value of 1·4 units.

TABLE III.—*Patients Suffering From Cancer of the Stomach*

| Case No. | Sex | Age | Urine Vol. (ml.) | pH | S.G. | β-glucuronidase Units/ml. |
|---|---|---|---|---|---|---|
| 1 | M. | 48 | 1560 | 5·7 | 10 | 0·45 |
| 2 | M. | 42 | 2380 | 6·1 | 03 | 0·70 |
| | | | 2450T | 6·4 | 04 | 0·50 |
| 3 | M. | — | 1100 | 7·0 | 20 | 0·60 |
| 4 | M. | 56 | 1080 | 5·8 | — | 0·84 |
| 5 | M. | 36 | 1470 | 5·8 | 12 | 1·3 |
| | | | 1930 | 6·7 | 12 | 1·6 |
| | | | 1600 | 6·0 | 09 | 1·1 |
| 6 | M. | 39 | 900 | 5·6 | — | 1·5 |
| 7 | M. | 65 | 1240 | 5·5 | 15 | 1·7 |
| | | | 1120 | 5·6 | 12 | 1·8 |
| 8 | M. | 54 | 1460 | 6·2 | 19 | 2·8 |
| | | | 1290 | 6·2 | 14 | 2·9 |
| | | | 2040 | 6·8 | 10 | 2·3 |

TABLE IV.—*Patients Suffering from Argentaffinoma*

| Case No. | Sex. | Age | Urine Vol (ml.) | pH | S.G. | β-glucuronidase Units/ml. |
|---|---|---|---|---|---|---|
| 1 | M. | — | 1800 | 6·4 | — | 0·7 |
| 2 | M. | 54 | 1900 | 7·9 | — | 0·8 |
| 3 | M. | — | — | 7·9 | — | 0·9 |
| 4 | M. | 70 | 2020 | 5·6 | 11 | 1·1 |
| | | | 1590 | 5·8 | 12 | 0·8 |
| | | | 2320T | 5·8 | 09 | 0·9 |
| | | | 1700T | 5·5 | 11 | 0·7 |
| 5 | M. | 65 | 735 | 6·4 | — | 1·0 |
| 6 | M. | 39 | 1025 | 7·3 | — | 3·2 |

TABLE V.—*Patients Suffering from Cancer of the Oesophagus*

| Case No. | Sex | Age | Urine Vol. (ml.) | pH | S.G. | β-glucuronidase Units/ml. |
|---|---|---|---|---|---|---|
| 1 | F. | 68 | 1890 | 4·8 | — | 0·30 |
| 2 | M. | 45 | 2050 | 5·8 | — | 0·80 |
| 3 | M. | 61 | 3320 | 5·8 | 08 | 1·1 |
| 5 | M. | 67 | 2220 | 5·8 | 09 | 1·0 |
| | | | 1640 | 5·8 | 08 | 1·4 |
| 5 | M. | 39 | 890 | 5·8 | 12 | 1·2 |
| 6 | M. | 51 | 1530T | 5·5 | 12 | 1·3 |
| 7 | M. | 61 | 370 | 5·5 | 24 | 4·2 |
| | | | 450 | 5·5 | 26 | 4·2 |

TABLE VI.—*Patients Suffering from Cancer of the Colon or Rectum*

| Case No. | Sex | Age | Urine Vol. (ml.) | pH | S.G. | β-glucuronidase Units/ml. |
|---|---|---|---|---|---|---|
| | | | (a) *Colon* | | | |
| 1 | M. | 66 | 1320 | 5·6 | — | 0·27 |
| 2 | F. | 53 | 890 | 6·4 | 12 | 0·46 |
| | | | 1130 | 6·4 | 08 | 0·28 |
| 3 | M. | 47 | 1800 | 5·4 | — | 0·54 |
| 4 | F. | 58 | 1550 | 5·2 | — | 0·65 |
| 5 | M. | 68 | 700 | 5·2 | 09 | 0·77 |
| | | | (b) *Rectum* | | | |
| 1 | M. | 55 | 1250 | 6·0 | 08 | 1·1 |
| 2 | M. | 72 | 1500 | 5·5 | 10 | 1·1 |
| | | | 1800 | 5·6 | 10 | 1·2 |
| 3 | M. | 65 | 1640 | 6·0 | 15 | 1·5 |
| | | | 1310 | 7·0 | 12 | 1·4 |
| 4 | M. | 45 | 2100 | 6·1 | 15 | 1·3 |
| | | | 1340 | 6·4 | 20 | 1·7 |
| | | | 2370 | 5·5 | 06 | 1·0 |
| 5 | M. | 72 | 1450 | 5·5 | 10 | 1·6 |

*Patients suffering from cancer of the larynx*

Of the 20 patients suffering with cancer of the vocal cord (Table VII) only 4 have β-glucuronidase activities within the normal range, with a mean 1·7 units. Of 11 patients suffering from cancer of the larynx but not cancer of the vocal cord (Table VIII), 5 had values above the range of healthy subjects, the mean value being 1·6 units.

TABLE VII.—*Patients Suffering from Cancer of the Larynx*

(a) *Cancer of the Vocal Cord*

| Case No. | Sex | Age | Urine Vol (ml.) | pH | S.G. | β-glucuronidase units/ml. |
|---|---|---|---|---|---|---|
| 1 | M. | 50 | 2750 | 5·5 | 10 | 0·60 |
| 2 | M. | 52 | 1210T | 5·5 | 10 | 0·60 |
| | | | 1100 | 5·5 | 08 | 0·70 |
| 3 | M. | 84 | 2120 | 5·5 | 11 | 1·2 |
| | | | 2290 | 5·5 | 12 | 1·0 |
| 4 | M. | 68 | 2630 | 6·7 | 0·4 | 0·8 |
| | | | 2380T | 6·8 | 0·5 | 1·6 |
| 5 | M. | 63 | 1330 | 6·0 | 14 | 1·5 |
| | | | 2590T | 7·2 | 07 | 1·0 |
| 6 | M. | 61 | 2300 | 6·7 | 10 | 1·5 |
| | | | 1490T | 5·5 | 10 | 1·0 |
| 7 | M. | 60 | 1010 | — | — | 1·4 |
| 8 | F. | — | 650 | 5·4 | — | 1·5 |
| 9 | M. | 58 | 1770 | 5·8 | 21 | 1·5 |
| | | | 2060 | 5·9 | 08 | 1·5 |
| 10 | M. | 67 | 710 | 7·0 | 18 | 1·6 |
| | | | 1330T | 6·4 | 10 | 1·5 |
| 11 | M. | 35 | 1700 | 5·5 | 19 | 1·7 |
| | | | 1910T | 5·6 | 12 | 1·3 |
| 12 | M. | 67 | 1130 | 5·5 | 14 | 1·7 |
| 13 | M. | 60 | 710 | 6·0 | 29 | 1·5 |
| | | | 540T | 5·5 | 15 | 2·3 |
| 14 | M. | 64 | 1080T | 5·8 | 17 | 1·8 |
| 15 | M. | 61 | 2100 | 6·1 | 11 | 1·7 |
| | | | 2320T | 5·5 | 10 | 1·6 |
| | | | 660 | 5·8 | 15 | 2·5 |
| 16 | M. | 38 | 910T | 5·8 | 15 | 2·4 |
| | | | 710 | 7·0 | 18 | 1·6 |
| 17 | F. | 61 | 2500 | 5·8 | — | 2·9 |
| | | | 1600 | 6·4 | 11 | 1·1 |
| 18 | M. | 55 | 740 | 5·6 | 23 | 2·5 |
| | | | 850T | 5·5 | 28 | 3·1 |
| 19 | M. | 83 | 1270 | 7·5 | 10 | 3·4 |
| | | | 1260 | 7·3 | 13 | 3·7 |
| 20 | M. | 73 | 890 | 6·1 | 18 | 4·1 |
| | | | 900T | 5·6 | 14 | 3·2 |

TABLE VIII.—*Patients Suffering from Cancer of the Larynx*

(b) *Cancer of Sites other than Vocal Cord*

| Case No. | Sex | Age | Urine Vol. (ml.) | pH | S.G. | β-glucuronidase Units/ml. |
|---|---|---|---|---|---|---|
| 1 | M. | 69 | 2350T | 5·5 | 10 | 0·6 |
| 2 | M. | 61 | 2340T | 6·1 | 06 | 0·7 |
| 3 | M. | 57 | 2660 | 7·0 | 06 | 1·0 |
| | | | 2520T | 7·0 | 14 | 0·7 |
| 4 | M. | 56 | 2550T | 6·7 | 10 | 1·0 |
| 5 | F. | 84 | 940 | 6·0 | 10 | 1·0 |
| | | | 1530T | 5·8 | 05 | 1·2 |
| 6 | M. | 39 | 890 | 5·8 | 12 | 1·2 |
| 7 | M. | 51 | 1530T | 5·5 | 12 | 1·3 |
| 8 | M. | 64 | 2590T | 6·4 | 09 | 1·6 |
| 9 | M. | 62 | 1720T | 5·8 | 07 | 1·7 |
| 10 | M. | 54 | 1370T | 6·9 | 18 | 2·5 |
| 11 | M. | 57 | 860T | 7·0 | 15 | 2·7 |

*Patients suffering from cancer of the bronchus*

Of 11 patients suffering from cancer of the bronchus (Table IX) 5 cases have a β-glucuronidase activity within the normal range and the mean is 1·8 units.

TABLE IX.—*Patients Suffering from Cancer of the Bronchus*

| Case No. | Sex | Age | Urine Vol. (ml.) | pH | S.G. | β-glucuronidase Units/ml. |
|---|---|---|---|---|---|---|
| 1 | F. | 72 | 1700 | 6·0 | 10 | 0·50 |
| | | | 1090 | 5·5 | 14 | 0·90 |
| 2 | M. | 49 | 2270 | 6·1 | 16 | 0·60 |
| | | | 2800T | 7·0 | 06 | 1·1 |
| 3 | M. | 75 | 500 | — | — | 1·0 |
| 4 | M. | 57 | 1720 | 5·5 | 12 | 1·0 |
| | | | 860 | 5·8 | 13 | 1·2 |
| 5 | M. | 62 | 720 | 6·8 | 12 | 0·8 |
| | | | 1200 | 6·0 | — | 1·5 |
| 6 | M. | — | 980 | 6·1 | 10 | 1·6 |
| 7 | M. | 66 | 1230 | 6·4 | 21 | 1·7 |
| | | | 1980 | 6·4 | 24 | 1·8 |
| 8 | M. | 62 | 720 | 6·8 | 12 | 0·8 |
| | | | 1200 | 6·0 | — | 1·5 |
| 9 | M. | 69 | 1400T | 6·4 | 15 | 2·4 |
| 10 | M. | 63 | 1400 | 7·0 | 14 | 2·4 |
| 11 | M. | 56 | 870 | 6·8 | 14 | 4·8 |
| | | | 1370 | 6·8 | 14 | 4·9 |

*Patients suffering from malignant blood diseases*

The urinary β-glucuronidase activity of 8 out of 10 patients suffering from leukaemia (Table X*a*) was above the normal range with an average activity of 1·8 units. Cases 9 and 10 had pyrexia at the time of collection. Of 5 patients suffering from Hodgkin's disease (Table X*b*) 4 had activity above the normal range and the average value is 2·5 units.

TABLE X.—*Patients Suffering from Malignant Blood Diseases*

| Case No. | Sex | Age | Urine Vol. (ml.) | pH | S.G. | β-glucuronidase Units/ml. |
|---|---|---|---|---|---|---|
| | | | (a) *Leukaemia* | | | |
| 1 | M. | 44 | 1500 | 6·0 | — | 1·5 |
| 2 | M. | 24 | 2200 | 5·7 | — | 1·2 |
| 3 | M. | 50 | 2500 | 6·7 | 09 | 1·2 |
| | | | 2100 | 6·7 | 11 | 0·98 |
| | | | 2500 | 5·8 | 67 | 1·9 |
| 4 | F. | 71 | 1850 | 7·0 | 10 | 1·7 |
| | | | 1860T | 7·0 | 09 | 1·2 |
| 5 | F. | 76 | 1760 | 6·2 | — | 1·5 |
| 6 | F. | 44 | 1500 | 6·0 | — | 1·5 |
| 7 | M. | 70 | 1440 | 6·7 | 12 | 1·9 |
| 8 | F. | 16 | 1630 | 7·0 | 20 | 2·7 |
| | | | 1410T | 7·4 | 24 | 2·4 |
| 9 | F. | 26 | 1680 | 5·8 | 09 | 3·0 |
| 10 | M. | 38 | 2620 | 7·0 | 10 | 3·4 |
| | | | (b) *Hodgkins Disease* | | | |
| 1 | M. | 34 | 1890 | 6·4 | 07 | 0·50 |
| | | | 1890 | 6·0 | 08 | 0·70 |
| 2 | M. | 48 | 720 | 5·8 | 20 | 1·3 |
| | | | 1630 | 6·4 | 15 | 2·7 |
| 3 | M. | 42 | 2800 | 5·8 | 05 | 3·0 |
| | | | 1800 | 6·1 | 12 | 2·8 |
| | | | 1200 | 6·0 | 10 | 2·7 |
| 4 | F. | 50 | 420T | 5·8 | 28 | 3·2 |
| 5 | M. | 63 | 1440 | 5·5 | 20 | 3·6 |
| | | | 710 | 7·0 | — | 3·8 |

*Patients suffering from cancer of the breast*

Of 15 patients suffering from cancer of the breast (Table XI), 8 are above the normal range of activity. Case 5 is a male with cancer of the breast. The average $\beta$-glucuronidase activity was 1·5 units.

TABLE XI.—*Patients Suffering from Cancer of the Breast*

| Case No. | Age | Urine Vol. (ml.) | pH | S.G. | β-glucuronisdase Units/ml. |
|---|---|---|---|---|---|
| 1 | 55 | 1250 | 6·7 | 09 | 0·3 |
| 2 | 52 | 2500 | 5·8 | — | 0·8 |
| 3 | 47 | 1820 | 5·5 | — | 1·0 |
| 4 | 48 | 2400 | 6·9 | 10 | 1·2 |
| 5 | 42 | 1910T | 5·5 | 12 | 1·2 |
| 6 | 55 | 1200 | 5·5 | — | 1·2 |
| 7 | 55 | 1750 | 6·2 | — | 1·2 |
| 8 | 62 | 1570 | 4·8 | — | 1·5 |
| 9 | 53 | 1820 | 7·0 | 08 | 1·6 |
| 10 | 61 | 2000 | 6·5 | — | 1·9 |
| | | 1860 | 9·3 | 13 | 1·3 |
| 11 | 48 | 1140 | 6·0 | 18 | 2·2 |
| | | 1800T | 5·8 | 14 | 1·3 |
| 12 | 38 | 1375 | 6·5 | — | 1·8 |
| 13 | 78 | 2020 | — | — | 2·2 |
| 14 | 54 | 960 | 4·5 | — | 2·5 |
| 15 | 63 | 1200 | 5·6 | 14 | 2·6 |
| | | 1030T | 5·6 | 12 | 2·5 |

*Patients suffering from cancer of the testicle*

Of 15 patients suffering from cancer of the testicle (Table XII) 10 have $\beta$-glucuronidase values above the normal range. The average $\beta$-glucuronidase value is 1·5 units.

TABLE XII.—*Patients Suffering from Cancer of the Testicle*

| Case No. | Age | Urine Vol. (ml.) | pH | S.G. | β-glucuronidase Units/ml. |
|---|---|---|---|---|---|
| 1 | 29 | 1800 | — | — | 0·80 |
| 2 | 39 | 1400 | 5·4 | — | 0·85 |
| 3 | 45 | 610 | 5·8 | 24 | 1·0 |
| 4 | 35 | 1750 | 6·5 | — | 1·0 |
| 5 | 55 | 1830 | 8·5 | 11 | 1·2 |
| 6 | 37 | 2190 | 7·6 | 14 | 0·8 |
| | | 2000T | 7·0 | 12 | 1·6 |
| 7 | 45 | 2100 | 6·1 | 15 | 1·3 |
| | | 1340 | 6·4 | 20 | 1·7 |
| | | 2370 | 5·5 | 06 | 1·0 |
| 8 | 18 | 1500 | 5·5 | — | 1·5 |
| | | 900 | 6·0 | — | 1·3 |
| 9 | 31 | 2100 | 7·0 | — | 1·4 |
| | | 2100 | — | — | 1·4 |
| | | 2200 | 6·5 | — | 1·6 |
| 10 | 31 | 2100 | 7·0 | — | 1·4 |
| 11 | 18 | 1500 | 5·4 | — | 1·5 |
| 12 | 30 | 1500 | 5·5 | — | 2·5 |
| | | 2550 | 5·8 | — | 1·2 |
| 13 | 68 | 1270 | 5·5 | 10 | 2·5 |
| | | 1330 | 5·5 | 10 | 2·2 |
| | | 1730 | 5·5 | 12 | 2·0 |
| 14 | 39 | 1250 | 5·9 | 18 | 1·8 |
| | | 450T | 5·5 | 12 | 4·0 |
| 15 | 29 | 1080 | — | 16 | 4·2 |
| | | 940 | — | 18 | 1·7 |

*Patients suffering from cancer of the prostate*

(a) *Untreated patients.*—Of 14 patients with cancer of the prostate examined before treatment (Table XIII) one-half have values above normal range. The average β-glucuronidase activity is 1·5 units/ml.

TABLE XIII.—*Patients Suffering from Cancer of the Prostate*

(a) *Untreated Patients*

| Case No. | Age | Urine Vol. (ml.) | pH | S.G. | β-glucuronidase Units/ml. |
|---|---|---|---|---|---|
| 1 | 75 | 1700 | 6·0 | — | 0·55 |
| 2 | 67 | 1700 | 5·5 | 12 | 0·79 |
| 3 | 51 | 1850 | 6·1 | 08 | 0·95 |
| 4 | 62 | 1250 | 5·6 | — | 1·0 |
| 5 | 58 | 1920 | 6·5 | 12 | 1·0 |
| 6 | 79 | 1060 | 5·2 | 17 | 1·3 |
| | | 2000 | 5·2 | 08 | 1·0 |
| | | 1500 | 5·2 | 12 | 1·2 |
| 7 | 66 | 1100 | — | — | 1·1 |
| 8 | 68 | 1720 | 5·8 | 10 | 0·5 |
| | | 1490 | 6·0 | 08 | 1·0 |
| | | 1100 | 5·8 | 10 | 1·3 |
| | | 1170T | 6·7 | 15 | 2·7 |
| 9 | 70 | 1910 | 5·7 | 13 | 1·2 |
| | | 1995 | 5·8 | 14 | 1·3 |
| 10 | 57 | 2600 | — | — | 1·4 |
| 11 | 63 | 620 | 5·5 | 20 | 2·2 |
| | | 620T | 5·5 | 22 | 1·3 |
| 12 | 49 | 1290 | 7·0 | 10 | 2·3 |
| | | 1440T | 7·1 | 11 | 1·9 |
| 13 | 70 | 1230 | 5·5 | 12 | 2·9 |
| | | 1640 | 5·5 | 09 | 2·6 |
| | | 1940 | 5·8 | 10 | 3·8 |
| 14 | 66 | 1140 | 7·0 | 06 | 2·9 |

(b) *Patients treated with stilboestrol for more than two years.*—The mean β-glucuronidase activity of the urine of a group of 7 patients receiving treatment with stilboestrol for more than two years (Table XIV) is 2·3 units and all were above the normal range.

TABLE XIV.—*Patients Suffering from Cancer of the Prostate*

(b) *Patients Treated with Stilboestrol for more tham Two Years*

| Case No. | Age | Urine Vol. (ml.) | pH | S.G. | β-glucuronidase Units/ml. |
|---|---|---|---|---|---|
| 1 | 70 | 2070 | 5·5 | 13 | 1·6 |
| | | 2170 | 7·0 | 13 | 1·5 |
| 2 | 69 | 1100 | 6·1 | 21 | 1·8 |
| | | 1930 | 5·7 | 11 | 1·8 |
| 3 | 73 | 1590 | 6·9 | 20 | 2·0 |
| | | | 6·9 | 13 | 1·5 |
| 4 | 66 | 970 | 5·8 | 15 | 2·2 |
| | | 1020 | 5·8 | 13 | 2·0 |
| 5 | 72 | 2580 | 2·3 | 12 | — |
| | | 1570 | 6·4 | 20 | 2·6 |
| 6 | 68 | 1360 | 7·0 | 15 | 2·9 |
| | | 1020 | 7·0 | 19 | 3·2 |
| 7 | 69 | 1650 | 7·0 | 17 | 2·9 |
| | | 1400 | 7·0 | 15 | 3·3 |

*Patients suffering from cancer of the thyroid*

The results obtained from 7 patients with cancer of the thyroid (Table XV) show only two of these patients had $\beta$-glucuronidase activities greater than the upper limit of normal and in both of these cases the patients had raised temperatures at the time of collection of the specimen. The average $\beta$-glucuronidase activity of this group was 1·1 units.

TABLE XV.—*Patients Suffering from Cancer of the Thyroid*

| Case No. | Sex | Age | Urine Vol. (ml.) | pH | S.G. | β-glucuronidase Units/ml. |
|---|---|---|---|---|---|---|
| 1 | F. | 57 | 1850 | 5·5 | — | 0·35 |
| | | | 1800 | — | — | 0·36 |
| | | | 1900 | 5·8 | — | 0·31 |
| 2 | F. | 70 | 2500 | 6·0 | — | 0·45 |
| | | | 1750 | 6·1 | — | 0·58 |
| | | | 2300 | 6·0 | — | 0·38 |
| 3 | M. | 48 | 2120 | 7·0 | 09 | 0·35 |
| | | | 1900 | 6·7 | 11 | 0·60 |
| 4 | F. | 68 | 1800 | 5·3 | — | 0·50 |
| | | | 2050 | — | — | 0·52 |
| | | | 2010 | 5·6 | 15 | 0·50 |
| 5 | F. | — | 1500 | 6·1 | 18 | 0·80 |
| | | | 1675 | 6·1 | 22 | 0·50 |
| 6 | F. | — | 1130 | 6·7 | 19 | 1·8 |
| | | | 980 | 6·7 | 09 | 1·6 |
| 7 | F. | 68 | 570 | 7·0 | 15 | 3·4 |
| | | | 500 | 6·4 | 19 | 4·1 |

### DISCUSSION

The $\beta$-glucuronidase excretion has been expressed as activity per unit volume of urine but in all cases the volume of the 24-hour specimens of urine have also been recorded so that total excretions of $\beta$-glucuronidase per 24 hours may be obtained. The method of estimation has been modified so that even alkaline urine specimens, previously discarded may now estimated, but in very alkaline conditions the enzyme is not stable.

The urinary $\beta$-glucuronidase activities of patients varied considerably with the site of the tumour but in no group of cases were all the results outside the normal range. This suggests that $\beta$-glucuronidase activity could not be used as a diagnostic method, although in some types of cancer such estimations might prove useful for prognostic purposes.

The average urinary $\beta$-glucuronidase activity in cancer of various sites within the alimentary tract is, in general, near the upper limit of normal but the activity in cases of cancer of the colon is low. Cancer of the thyroid also produced a decrease in urinary $\beta$-glucuronidase activity but cases of cancer of larynx, bronchus, oesophagus, prostate and testicle show an increased activity.

Cases of leukaemia and of Hodgkin's disease also exhibit an increase in urinary $\beta$-glucuronidase activity but in these cases there is generally an increase in the patient's temperature, which is known to increase $\beta$-glucuronidase excretion (Boyland and Williams, unpublished), so that the increased activity may be due partly to this cause. The connection between malignant blood disease and blood glucuronidase activity and the treatment of these patients with enzyme inhibitors is being investigated.

SUMMARY

(1) The method previously used for the estimation of urinary $\beta$-glucuronidase activity has been modified so that it may be applied to alkaline or concentrated urine.

(2) Urinary $\beta$-glucuronidase activities have been investigated in patients suffering from cancer of the alimentary tract, larynx, breast, thyroid, bronchus, testicle, and prostate and also in patients suffering from leukaemia and Hodgkin's disease.

We should like to thank Professor F. C. Ormerod of the Institute of Laryngology; Dr. L. M. Franks of the Imperial Cancer Research Fund Laboratories, Royal College of Surgeons; and Dr. M. Sandler of the Royal Free Hospital for the collection of specimens and Mr. J. W. Gorrod for technical assistance.

This investigation has been supported by grants to the Chester Beatty Research Institute (Institute for Cancer Research; Royal Cancer Hospital) from the British Empire Cancer Campaign, the Jane Coffin Childs Memorial Fund for Medical Research, the Anna Fuller Fund and the National Cancer Institute of the National Institutes of Health, U.S. Public Health Service.

REFERENCES

ANLYAN, A. J. AND STARR, A.—(1952) *Cancer*, **5**, 578.

BOYLAND, E., WALLACE, D. M. AND WILLIAMS, D. C.—(1955*a*) *Brit. J. Cancer*, **9**, 62.—(1955*b*) *Brit. J. Urol.*, **27**, 11.

*Idem* AND WILLIAMS, D. C.—(1956) *Biochem. J.*, **64**, 578.

FISHMAN, W. H.—(1947) *Science*, **105**, 646.

*Idem* AND ANLYAN, A. J.—(1947*a*) *Ibid.*, **106**, 66.—(1947*b*) *J. biol. Chem.*, **169**, 449.

*Idem*, ANLYAN, A. J. AND GORDON, E.—(1947*d*) *Cancer Res.*, **7**, 808.

*Idem*, KASDON, S. C. AND HOMBURGER, F—(1950) *J. Amer. med. Ass.* **143**, 350.

*Idem*, MARKUS, R. L., PAGE, O. C., PFEIFFER, P. H. AND HOMBURGER, F.—(1950) *Amer. J. med. Sci.*, **220**, 55.

KERR, L. M. H. AND LEVVY, G. A.—(1947) *Nature*, **160**, 463.

LEVVY, G. A., KERR, L. M. H. AND CAMPBELL, J. G.—(1948) *Biochem. J.* **41**, 462.

McDONALD, D. F. AND ODELL, L. D.—(1947) *J. clin. Endocrin.*, **7**, 535.

ODELL, L. D. AND McDONALD, D. F.—(1948) *Amer. J. Obstet. Gynec.*, **56**, 1.

TALALAY, F., FISHMAN, W. H. AND HUGGINS, C.—(1946) *J. biol. Chem.*, **166**, 757.